# Using a Scorecard to Track Neurological Care Disparities Related to Clinical Decision-Making: A Proof of Concept and Call to Action

**DOI:** 10.7759/cureus.92781

**Published:** 2025-09-20

**Authors:** Roshni Dhoot, Deborah Rose, Shakthi Unnithan, Danelvis Paredes, Meenakshi Roy, Charles H Roark, Petra Brayo, Hussein Al-Khalidi, Andrew R Spector

**Affiliations:** 1 Neurology, Duke University Medical Center, Durham, USA; 2 Neurology, Johns Hopkins Health System, Baltimore, USA; 3 Biostatistics, Duke University Medical Center, Durham, USA; 4 Neurology, Northeast Georgia Medical Center Gainsville, Gainsville, USA; 5 Neurology, Rush University Medical Center, Chicago, USA; 6 Neurology, Parkridge Health System, Chattanooga, USA; 7 Neurology, Temple University Hospital, Philadelphia, USA

**Keywords:** disparities, ethnicity, implicit bias, race, scorecard

## Abstract

Introduction

Health disparities result from a complex interplay of factors, with social determinants of health and inequities in healthcare delivery playing central roles. Among these, clinicians have a significant influence on rectifying healthcare-related causes. However, many clinicians remain unaware of their potential contributions to these disparities because individual clinical practices are seldom scrutinized to identify departmental or practitioner-level disparities. This study presents a model developed by an academic neurology department to systematically track and address disparities in clinical care, offering a replicable approach for other departments.

Methods

To develop a scorecard, eight clinical divisions were selected, each with one binary clinical decision point analyzed. The percentage of patients who received the designated intervention was stratified by race/ethnicity to identify any disparities in care. The presence or absence of a racial disparity in the care provided was translated into a scorecard for easy communication of results. The percentage of patients who received the designated intervention was stratified by race and ethnicity to identify any disparities in care. Findings were translated into a scorecard format, assigning grades based on the presence or absence of statistically significant disparities.

Results

The department’s scorecard contained five As and two Bs (and one division with inadequate data) based on disparities of care identified in two divisions, stroke and epilepsy. The department’s scorecard revealed that five divisions received an “A” rating, indicating no significant racial or ethnic disparities. One division had insufficient data for grading.

Conclusion

Scorecards are a practical tool for identifying and addressing disparities in clinical care. This information can facilitate transparency, promote targeted practice improvements, and strengthen trust with patients and communities. By adopting scorecards, institutions can enhance equity in care delivery and build credibility through accountability.

## Introduction

Racial and ethnic disparities in neurological disorders are well-documented. For example, Black Americans have a 45% higher stroke mortality risk than non-Hispanic White Americans [1], exhibit greater cognitive impairment and neuropsychiatric symptoms in dementia [2], and have more severe symptoms and disability from multiple sclerosis [3].

Identifying the existence of a disparity is a critical first step, but it does not uncover the causes. Social and structural drivers of health, such as exposure to pollution, lack of educational opportunities, unsafe housing, and racial discrimination, loom large in differential burdens of disease and less favorable outcomes [4]. Differences in the delivery of healthcare can also lead to disparities [5]. Improving health equity requires understanding its causes, including when providers propagate the inequities. Local and individual clinical practice patterns can be analyzed to reveal specifics that national datasets cannot capture. Data must then be provided back to clinicians in a convenient fashion, such as a scorecard [6,7].

Scorecards are utilized by multiple organizations and government agencies to describe disparities between racial and ethnic groups [8]. National scorecards are unable to identify concerns about individual clinical practices, though. Local scorecards, on the other hand, have been shown to improve clinical outcomes [6], and it has been suggested that clinicians should have ready access to their health equity data [7]. Furthermore, improving transparency about racial disparities or, ideally, the lack of racial disparities, can help build back credibility in the local healthcare system among marginalized groups for whom distrust can be an obstacle to optimal care [9].

Inspired by the Racial Justice Report Card published by White Coats for Black Lives [10], which evaluates medical schools on markers of anti-racism, such as recruitment, curriculum, and provisions of health care, we developed a racial equity clinical scorecard to reflect disparities in care within our institution’s neurology department. This paper describes the development and implementation of the scorecard, providing a model for other institutions to track and address such disparities.

## Materials and methods

Standard protocol approvals, registrations, and patient consents

The Duke University Health System (DUHS) Institutional Review Board (IRB) has determined that the following protocol meets the criteria for a declaration of exemption from further IRB review as described in 45 Code of Federal Regulations (CFR) 46.101(b), 45 CFR 46.102 (f), or 45 CFR 46.102 (d), satisfies the privacy rule as described in 45 CFR 164.512(i), and satisfies Food and Drug Administration regulations as described in 21 CFR 56.104, where applicable.

Study population

Records were reviewed if they included a date of service by a Neurology clinician (resident, fellow, advanced practice provider, or attending) from January 1, 2020, to December 31, 2021. Records were subdivided based on International Statistical Classification of Diseases and Related Health Problems, Tenth Revision (ICD-10) diagnostic codes associated with the neurology encounter(s) [11]. Multiple clinical encounters by the same patient were analyzed together, such that if an intervention were offered at any of the visits, it would count as having been offered to the patient. Encounter types with any of the following descriptions were included in the analysis for all clinical decisions other than for stroke: initial consults, office visits, telemedicine, telemedicine-phone, and refills. For stroke, only hospital encounter types were retained. 

Clinical decision-making points

The following decision points were analyzed to grade each division: the administration of alteplase for stroke [1]; referral to psychology for insomnia [12]; prescription for high-efficacy disease-modifying therapy (DMT) for multiple sclerosis (MS), defined as any of the following: ofatumumab, natalizumab, alemtuzumab, ocrelizumab, fingolimod, siponimod, cladribine, or ozanimod [13]; prescription of erenumab, fremanezumab, galcanezumab, or eptinezumab for migraine [14]; prescription of azathioprine, mycophenolate, cyclosporine, tacrolimus, or eculizumab for myasthenia gravis (high-efficacy DMTs available in 2020) [15]; referral to neurosurgery for Parkinson’s disease [16]; referral to neurosurgery for temporal lobe epilepsy [17,18]; and prescription of donepezil, rivastigmine, galantamine, or memantine for Alzheimer’s disease (AD) [19].

These clinical decisions were based on two criteria: (1) existing national data demonstrating race-based differences in neurologic care, or (2) a determination by the authors that the decision point was at risk for exhibiting race-based disparities. For decisions in the latter category, clinical questions were reviewed by attending neurologists with subspecialty expertise to ensure both medical appropriateness and relevance.

Clinical decisions were reviewed retrospectively using data from the electronic medical record. Patient data was collected by the Duke Health Analytics Center of Excellence to ensure data quality and consistency. Statistical analysis was conducted by expert statisticians at the Duke Biostatistics, Epidemiology, and Research Design Core. Diagnoses were mapped to diagnostic codes from the ICD-10, third edition [11]. 

Stroke

Eligibility for inclusion included patients who arrived at the Duke University Hospital emergency department with an acute stroke code activation. Exclusion criteria consisted of charts with codes S06 (intracranial injury), I60 (non-traumatic subarachnoid hemorrhage), I61 (non-traumatic intracerebral hemorrhage), I62 (other non-traumatic intracranial hemorrhage), and American Medical Association current procedural terminology (CPT) code 61645 (cerebral endovascular therapy on skull, meninges, or brain). This was to exclude hemorrhagic strokes (not alteplase candidates) and those who received mechanical thrombectomy. Patients were not excluded based on presentation time from symptom onset, as the decision of whether or not to administer thrombolytics is ultimately at the discretion of the clinician. The intervention selected was a prescription for alteplase.

Epilepsy

All patients seen by an epilepsy neurologist or general neurologist for diagnosis code G40.2 (localization-related symptomatic epilepsy) were included. No exclusion criteria were applied. The intervention selected was a referral to neurosurgery with referral code G40.2.

Headache

All patients seen by a headache neurologist or general neurologist for diagnosis code G43.0 (migraine), G43.1 (migraine with aura), G43.4 (hemiplegic migraine), G43.7 (chronic migraine without aura), G3.8 (other migraine), and G43.9 (unspecified migraine) were included. No exclusion criteria were applied. The intervention selected was a prescription for any one or more of the following: erenumab, fremanezumab, galcanezumab, or eptinezumab.

Neuromuscular

All patients seen by a Duke neuromuscular specialist or general neurologist for diagnosis code of G70.00 (myasthenia gravis) were included. No exclusion criteria were applied. The intervention selected was a prescription for any of the following: azathioprine, mycophenolate mofetil, cyclosporine, tacrolimus, or eculizumab (the high efficacy DMTs available in 2020).

Neuroimmunology

All patients seen by a neuroimmunologist or general neurologist for diagnosis code G35 (multiple sclerosis) were included. Patients who were not prescribed any DMT were excluded. The intervention selected was a prescription for a high-potency therapy, defined as ofatumumab, natalizumab, alemtuzumab, ocrelizumab, fingolimod, siponimod, cladribine, and ozanimod.

Memory

All patients seen by a memory disorders neurologist or general neurologist for diagnosis code G30 (Alzheimer’s disease) were included. Exclusion criteria included patients with codes R00.1 (bradycardia) or I45 (other conduction disorders), as these conditions are contraindications to acetylcholinesterase inhibitors. The intervention selected was a prescription for donepezil, rivastigmine, galantamine, or memantine.

Movement Disorders

All patients seen by a movement disorders neurologist or a general neurologist for diagnosis code G20 (Parkinson’s disease) were included. Patients with additional codes representing cognitive disorders were excluded as these pose a contraindication to neurosurgical treatment of Parkinson’s disease: F01 (vascular dementia), F02 (dementia in other disease classified elsewhere), F03.90 (unspecified dementia), F05 (delirium due to known physiological condition), F10 (mental and behavioral disorders due to use of alcohol), G30 (Alzheimer’s disease), and G31 (other degenerative disease of the nervous system). The intervention was a referral to neurosurgery linked to diagnostic code G20 (Parkinson’s disease).

Sleep

All patients seen by a sleep or general neurologist for diagnosis codes F51.0 (insomnia not due to a substance or physiological condition) and G47.0 (disorders of initiating and maintaining sleep) were included. Exclusion criteria included known cognitive disorder which would indicate a poor candidate for cognitive behavioral therapy for insomnia (CBT-I): F03.90 (unspecified dementia), G30 (Alzheimer’s disease), G31 (other degenerative conditions of the nervous system), F01 (vascular dementia), F10 (alcohol-related disease), F05 (delirium due to known physiological condition), and F02 (dementia in other diseases classified elsewhere). The referral to Psychology was queried based on links to diagnostic codes F51.0 or G47.0.

Covariates

Race and ethnicity were self-identified by patients who were asked to choose from the options provided in the electronic medical record; we utilized their recorded responses. Patients were offered the following choices for race: American Indian or Alaskan Native, Asian, Asian Indian, Black or African American, Caucasian/White, Chinese, Filipino, Guamanian or Chamorro, Japanese, Korean, Native Hawaiian or Other Pacific Islander, Not reported/declined, Other, Samoan, Vietnamese. We use the terms “Black” and “White” to refer to the groups denoted in the medical record as “Black or African American” and “Caucasian/White,” respectively. Due to low numbers of patients who identify as a group other than White or Black, races included in the model were White, Black, and Other (including multiracial). Ethnicity was recorded as "Hispanic or "Not Hispanic." Records without a race or ethnicity selected were excluded. Insurance types were determined from the medical record and were grouped as “Commercial” (e.g. Blue Cross, liability, Managed Care, and workers comp), “federal government” (Medicare, Medicare Advantage, other government), “Medicaid” (Medicaid-pending, NC Medicaid, NC Medicaid Managed Care, out of state Medicaid, and special programs), and “Other” (any insurance types listed as other or special programs). All analyses controlled for insurance type.

Statistical analysis

Patient demographic and clinical characteristics were summarized as means [standard deviations (SDs)], medians (25th and 75th percentiles), minima and maxima for continuous variables and as counts (percentages) for categorical variables. Two models, generalized estimating equations (GEE) and mixed-effects logistic regression, were used. Both models included repeated measures for patients with a binary outcome (whether or not the patient received the intervention). GEE were used to assess the association of intervention outcome with race and ethnicity in separate models for epilepsy, headache, myasthenia gravis, neuroimmunology, memory, and stroke divisions. GEE models were fitted with a compound symmetric working correlation matrix, treating patients as repeated measures after adjusting for insurance type. Mixed-effects logistic regression model was used to assess the association between intervention outcomes with race and ethnicity, with adjustment for insurance type in Parkinson’s disease and sleep divisions, due to the imbalance in the number of patients who received the intervention in these divisions. Race, ethnicity, and insurance type were treated as fixed effects. Patients were a repeated measure with a variance-components correlation matrix. Patients who visited different neurology divisions remained in the respective models. Odds ratios (OR) with 95% confidence intervals (CI) and p-values were reported for all models (robust standard error estimates were used to construct the 95% CI in the mixed-effects logistic regression models). Statistical hypotheses were tested as two-sided at a 0.05 level of significance, and no adjustments were made for multiple comparisons. All statistical analysis was performed using SAS v9.4 (SAS Institute Inc., Cary, NC). No power analyses were conducted.

Scorecard grading system

Each division was assigned a letter grade based on the number of statistically significant disparities identified among racial and ethnic group dyads (White-Black, Black-Other, White-Other, and Not Hispanic-Hispanic). We used a chi-square test and considered p<0.05 significant. If no statistically significant disparities (p<0.05) were observed, a grade of “A” was assigned. Grades of “B,” “C,” “D,” and “F” corresponded to the number of disparities identified (1, 2, 3, or 4, respectively). The magnitude of the disparity was not included in the grading system. The grading system was deliberately left simple, as we wanted it to be easy to explain to our community members. The more variables that determined the grade (e.g., magnitude of each disparity), the more complicated it would be for the lay public to interpret.

## Results

Demographic characteristics

The initial dataset comprised 18,499 unique patients who were seen for any of the selected diagnostic codes between January 1, 2020, to December 31, 2021. After applying exclusion criteria to remove ineligible records, the final cohort included 16,099 patients. Table 1 provides demographic information about the entire cohort. Additional information about our methods, including the specific ICD-10 codes used, is available as a supplemental file.

**Table 1:** Patient Characteristics in the Overall Study Cohort Demographic characteristics of the study population, including age, sex, race, ethnicity, and insurance status. For categorical variables, values are presented as N (%), with the number of missing observations reported where applicable. For continuous variables, the mean (standard deviation), median, and interquartile range (Q1, Q3) are provided. Percentages are calculated using the number of non-missing observations as the denominator.

		Total (N=16,099)
Age at first encounter		
	Missing	8 (0.0%)
	Mean (SD)	54.7 (18.5)
	Median	55.0
	Q1, Q3	40.0-70.0
Sex		
	Missing	8 (0.0%)
	Female	10,368 (64.4%)
	Male	5,723 (35.6%)
Race		
	Missing	393 (2.4%)
	Asian	269 (1.7%)
	Black or African American	4035 (25.7%
	White	10,918 (69.5%)
	Other	484 (3.1%)
Ethnicity		
	Missing	564 (3.5%)
	Hispanic	613 (3.9%)
	Not Hispanic	14,922 (96.1%)
Insurance Status		
	Missing	885 (5.5%)
	Commercial	6,735 (44.3%)
	Federal Government	7,100 (46.7%)
	Medicaid	1,095 (7.2%)
	Other	284 (1.9%)

Clinical decision-making

No significant racial or ethnic disparities were identified in clinical decision-making for multiple sclerosis, migraine, myasthenia gravis, Parkinson’s disease (PD), or Alzheimer’s disease (Table 2). However, two statistically significant racial disparities were found in the stroke and epilepsy cohorts. For stroke, 3,332 encounters met the inclusion criteria, with 728 (21.8%) receiving alteplase. White patients had 31% higher odds of receiving alteplase compared to Black patients (95% CI: 1.03-1.66; p=0.03).

**Table 2:** Odds of Care Decisions by Race and Ethnicity Across Neurological Conditions Odds ratios (OR) and 95% confidence intervals (CI) are shown for the association between patient race/ethnicity and specific clinical decisions across multiple neurological conditions. For each condition, the reference group is noted in the “Effect” column. An OR greater than 1 indicates higher odds of receiving the specified care decision compared with the reference group; an OR less than 1 indicates lower odds. All models are adjusted for insurance type. Statistical significance was assessed at P<0.05.

Disease	Group	OR (95% CI)	P-value
Epilepsy			
	Black vs Other	2.78 (0.36, 21.54)	0.3269
	Black vs White	0.61 (0.25, 1.51)	0.2878
	Other vs White	0.22 (0.03, 1.48)	0.1191
	Hispanic vs Not Hispanic	4.86 (2.05, 11.56)	0.0003
Headache			
	Black vs Other	1.07 (0.81, 1.42)	0.6326
	Black vs White	0.94 (0.83, 1.08)	0.3972
	Other vs White	0.88 (0.68, 1.15)	0.3482
	Hispanic vs Not Hispanic	0.88 (0.66, 1.18)	0.3958
Myasthenia Gravis			
	Black vs Other	1.04 (0.49, 2.22)	0.9137
	Black vs White	0.93 (0.68, 1.28)	0.6491
	Other vs White	0.89 (0.44, 1.83)	0.7529
	Hispanic vs Not Hispanic	1.16 (0.5, 2.66)	0.7313
Neuroimmunology			
	Black vs Other	1.07 (0.26, 4.5)	0.9216
	Black vs White	1.08 (0.68, 1.72)	0.7527
	Other vs White	1 (0.25, 4.04)	0.9962
	Hispanic vs Not Hispanic	0.76 (0.2, 2.9)	0.6865
Memory			
	Black vs Other	0.65 (0.41, 1.03)	0.0658
	Black vs White	0.9 (0.73, 1.11)	0.3247
	Other vs White	1.38 (0.9, 2.1)	0.1367
	Hispanic vs Not Hispanic	1.56 (0.9, 2.73)	0.116
Stroke			
	Black vs Other	1.16 (0.59, 2.28)	0.674
	Black vs White	1.31 (1.03, 1.66)	0.0302
	Other vs White	1.13 (0.58, 2.21)	0.7223
	Hispanic vs Not Hispanic	0.94 (0.4, 2.16)	0.8763

In the epilepsy analysis, 8,417 encounters were included. Of these, 36 (0.4%) were referred to neurosurgery for evaluation. Non-Hispanic patients were roughly five times more likely than Hispanic patients to receive a referral to neurosurgery (OR=4.9, 95% CI:2.2, 11.6; p=0.003).

For sleep medicine, an insufficient number of referrals to Psychology for insomnia (46 referrals across 664 patients) prevented convergence of generalized estimating equation (GEE) models, precluding meaningful analysis. Similarly, low referral numbers for PD patients resulted in GEE model convergence issues. Mixed-effects models, which included race, ethnicity, and insurance type as fixed effects and patients as repeated measures, did not identify significant differences in referral patterns for PD.

Scorecard creation

**Figure 1:**
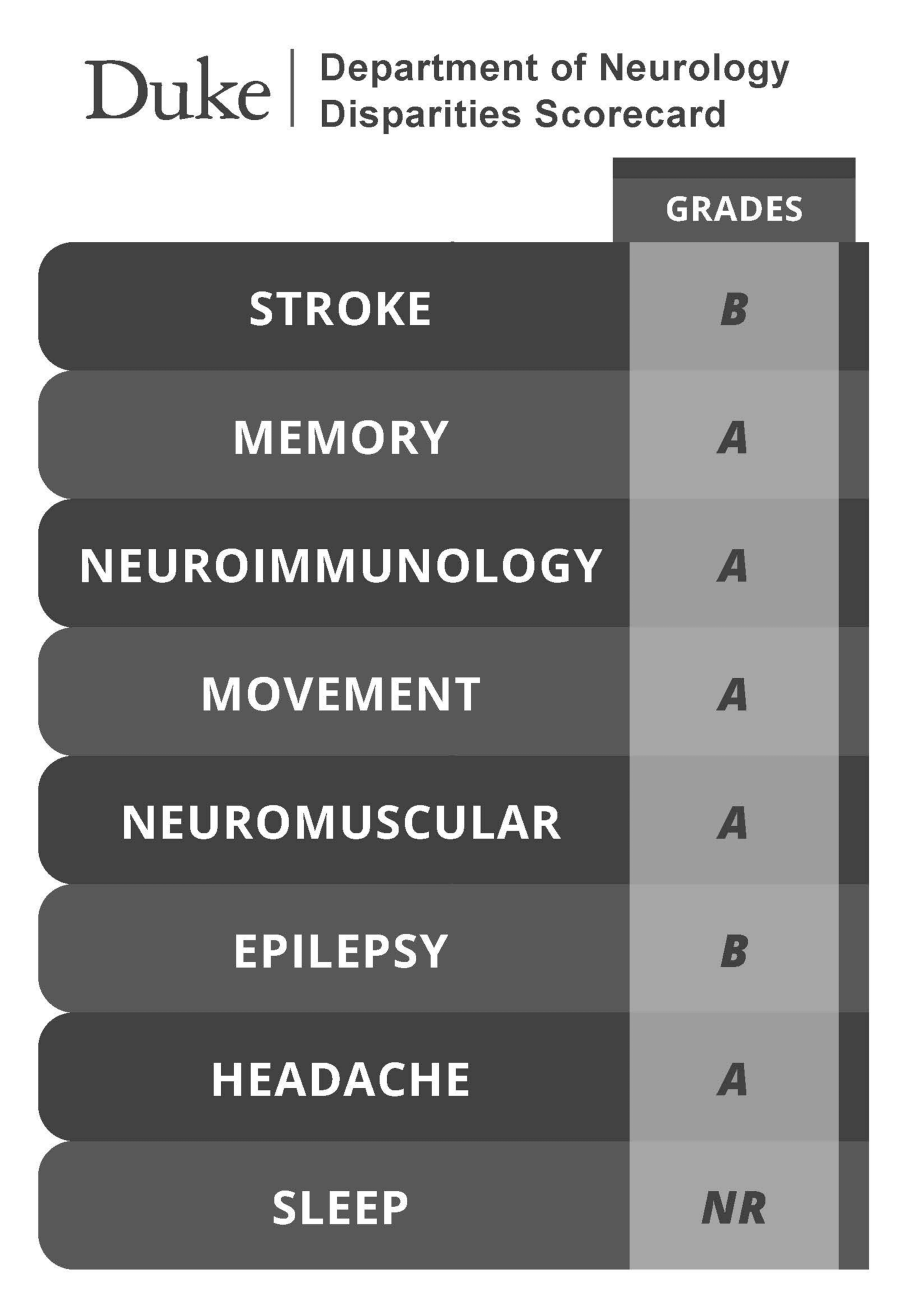
Duke Neurology Race and Ethnicity Disparities Scorecard Letter grades represent the number of statistically significant disparities identified in clinical decision-making across race and ethnicity for each neurological subspecialty. Grades were assigned as follows: A = 0 disparities, B = 1 disparity, C = 2 disparities, D = 3 disparities, F = 4 disparities. “Disparity” refers to a difference between any two racial or ethnic groups for the given decision that reached statistical significance. A total of eight specialties are shown; “NR” indicates that insufficient data were available to assign a grade (e.g., Sleep Medicine).

Based on the absence of racial or ethnic disparities in most of the clinical decisions studied, the scorecard (Figure 1) reflects predominantly grades of “A.” The two disparities noted occurred between two of the groups. In the case of stroke, there was a statistically significant White-Black disparity but not White-Asian, Black-Asian, or White-Hispanic, resulting in a grade of B. Similarly, a Hispanic-non-Hispanic disparity was found in epilepsy, also resulting in a grade of B.

## Discussion

The purpose of this study was to create a health disparities scorecard to display disparities results publicly. There are three primary benefits to this approach. First, the scorecard encourages departments to shift their focus internally for self-evaluation and mitigation of disparities at the clinician level. While multicenter research provides a broader view and is preferable for clinical trials, local analyses are essential to uncover practitioner-specific behaviors and inform targeted interventions. Personal awareness of disparities in clinical decisions has the potential to inspire behavioral changes among clinicians striving for equitable care [20]. 

A second benefit to the approach of a neurological health disparities scorecard is its practicality for tracking progress over time. Its flexibility allows departments to regularly update their assessments, ensuring ongoing accountability [10]. Third, the use of a disparities scorecard can improve relationships between medical centers and communities by increasing transparency [21]. Displaying a disparities scorecard on public platforms demonstrates a willingness to engage in conversation about healthcare racial and ethnic disparities, and actionable ways to address this issue; the scorecard (Figure 1) is currently displayed on our main departmental website. When the grades are high, it can provide reassurance to members of the community that the medical center is striving hard to provide unbiased care. This is one small step in repairing the credibility of health systems that have historically provided generations of inequitable, unjust, and discriminatory care disparity [22].

To facilitate the creation of scorecards for other departments, we also developed a user-friendly web calculator that will streamline the process [23]. Institutions can quickly generate their own scorecard by entering the number of patients who received a specific intervention and the total number eligible for it, stratified by each race or ethnic group. The web calculator can also be used for gender disparities or other groups. We strongly encourage institutions to publish these scorecards on public-facing websites to foster transparency and accountability while engaging stakeholders in conversation about healthcare equity. Identifying disparities at our institution highlights areas for targeted improvement over time.

Out of eight clinical decision points, our department identified racial and ethnic disparities in two areas - stroke and epilepsy - resulting in two grades of “B.” Specifically, White patients were significantly more likely than Black patients to receive alteplase for stroke, while non-Hispanic patients were more likely than Hispanic patients to be referred for neurosurgical evaluation in epilepsy. The remaining five divisions (multiple sclerosis, migraine, myasthenia gravis, Parkinson’s disease, and Alzheimer’s disease) received grades of “A,” indicating no significant disparities, while the sleep division could not be graded due to insufficient data.

For stroke, the disparity in alteplase administration between White and Black patients aligns with findings from prior national studies [1]. Potential explanations include implicit bias by the clinician at the time of clinical decision-making, systemic barriers, or differences in patient eligibility, such as time of presentation relative to the 4.5-hour treatment window. Importantly, this study could not determine if patients declined alteplase or were deemed ineligible, reinforcing the complexity of this issue.

In epilepsy, we found an ethnic but not racial disparity, while prior research has identified a Black-White disparity in surgery and not an ethnic disparity [17,18,24]. Disparities in referrals for neurosurgical evaluation between Hispanic and non-Hispanic patients raise concerns about equity in access to advanced treatment options. Clinicians play a pivotal role in ensuring that patients from all racial and ethnic backgrounds are fully informed of the potential benefits of surgical treatment. It is insufficient to attribute disparities solely to patient refusal, as counseling methods can heavily influence decision-making [25]. Thus, a patient’s decision to decline a neurosurgical referral may reflect how the option was presented rather than any inherent preferences or pre-existing concerns. This finding should prompt training in culturally responsive communication and equitable patient counseling among epilepsy clinicians.

A limitation of the epilepsy analysis is that some neurosurgical referrals may not have been documented in the electronic medical record, particularly those discussed informally between the neurologist and neurosurgeon during multidisciplinary conferences. While this would not account for the observed disparity by race and ethnicity, it contributes to the small number of documented referrals that were analyzed. Similarly, in sleep medicine, referrals to Psychology for CBT-I may have been underreported, particularly for patients referred to online CBT-I programs or external psychologists. Future research can use alternative methods to capture these patients for analysis. This could be accomplished by utilizing alternative medical records search strategies to find charts referencing CBT-I rather than relying on hard-coded referrals.

A potential limitation of our scorecard is that we rely on the number of disparities rather than the magnitude of each disparity. While this was done intentionally to keep the interpretation as straightforward as possible for our intended community audience, it restricts the information communicated by the scorecard.

The next step is to investigate the causes of these disparities and implement effective mitigation strategies. This study was not designed to answer that question, emphasizing the need for future research in this area.

## Conclusions

We developed a scorecard to systematically track and display neurological health disparities within an academic medical center. In this case, two disparities were identified, resulting in grades of mostly As and two Bs. The identified disparities can be studied further to reveal ways of increasing health equity. Scorecards offer a valuable tool for identifying and communicating disparities in a clear and actionable format. Making inequities visible at the departmental level facilitates targeted interventions and accountability. Departments that consistently achieve high grades can use scorecards to reinforce patient trust, demonstrating a commitment to equitable care, and strengthening institutional credibility.

With our online calculator, departments can generate customized scorecards for their specific patient populations. The tool can be used for any demographic variable, such as gender, primary language, race, ethnicity, and other social determinants of health. We encourage institutions to make their scorecards publicly available, reinforcing their commitment to equity and ensuring that disparities are actively addressed rather than overlooked.
